# Incidental learning and social‐communicative abilities in children with developmental language disorder: Further evaluating the implicit learning deficit hypothesis

**DOI:** 10.1111/1460-6984.70017

**Published:** 2025-02-20

**Authors:** Joseph H. R. Maes, Annette R. Scheper, Daan Hermans, Constance T. W. M. Vissers

**Affiliations:** ^1^ Donders Institute for Brain, Cognition and Behaviour Centre for Cognition Radboud University Nijmegen The Netherlands; ^2^ Royal Kentalis Utrecht The Netherlands; ^3^ Behavioral Science Institute Radboud University Nijmegen The Netherlands

**Keywords:** children, developmental language disorder, facial emotion recognition, implicit learning, language abilities, social‐communicative abilities

## Abstract

**Background:**

The implicit learning deficit hypothesis claims that impaired implicit learning underlies deficits in social‐communicative abilities associated with developmental language disorder (DLD). However, previous research testing this hypothesis revealed inconsistent results and largely used process‐impure sequential learning tasks.

**Aims:**

This study further tested the hypothesis using a novel process‐pure implicit associative learning task.

**Methods and Procedures:**

The performance of 9‐ to 13‐year‐old children with (*N* = 60) and without DLD (typically developing, TD, *N* = 52) on a contingency learning task (CLT) was compared. The task entailed the incidental learning of the contingency between simultaneously presented figure‐colour combinations. Also, the association of CLT performance with three aspects of social‐communicative abilities was assessed: facial emotion recognition ability, social responsiveness and language abilities.

**Outcomes and Results:**

Compared to the TD group, the DLD group performed equally on the CLT but showed worse performance on the measures of emotion recognition and social abilities. In neither group was CLT performance significantly related to any of the three social‐communicative abilities.

**Conclusions and Implications:**

These results do not support the implicit learning deficit hypothesis. The demonstrated intact implicit learning ability suggests the potential of using interventions to improve social‐communicative abilities in children with DLD that are based on incidental or implicit learning rather than on intentional or explicit learning.

**WHAT THIS PAPER ADDS:**

**What is already known on the subject**

- Individuals with developmental language disorder (DLD) not only have linguistic problems but also impaired abilities in social and emotional domains. According to the implicit learning deficit hypothesis, these impairments are largely due to a compromised ability to incidentally or implicitly learn regularities of stimuli or events occurring in daily life. However, research examining this hypothesis has yielded mixed results.

**What this paper adds to existing knowledge**

- This study contributes to the discussion about the presence or absence of a domain‐general implicit learning deficit in individuals with DLD, and corresponding potential clinical implications. For this, in addition to measures of language, emotion recognition and social abilities, a novel incidental learning task was used that was held to provide a more process‐pure measure of incidental and implicit learning compared to previous research. The collective results do not support the claim of a domain‐general implicit learning deficit underlying the social‐communicative and ‐emotional problems in DLD.

**What are the potential or actual clinical implications of this work?**

- Current interventions to improve language, social and emotional abilities in children with DLD are largely based on relatively effortful strategies involving explicit instruction and feedback. The present evidence of intact incidental and implicit learning abilities supports the examination of possible novel interventions that are based on relatively effortless implicit learning strategies, which may be more in line with how these abilities are acquired in daily life.

## INTRODUCTION

About seven in 100 children are diagnosed with developmental language disorder (DLD), formerly referred to as Specific Language Impairment (Bishop et al., 2017; Leonard, 2014; Tomblin et al., 2003, 1997). Compared to typically developing (TD) children, children with DLD have no known sensory, medical or intellectual deficits, but their language abilities are below average. The linguistic problems in DLD range from phonology and semantics to morphosyntax and pragmatics, both in language comprehension and production, and vary by age (Fey et al., 2004; Leonard, 2014).

Next to linguistic deficits, children with DLD also present more generally with problems in social‐communicative and social‐emotional domains (hereafter referred to as social‐communicative abilities), including emotion recognition (e.g., Bahn et al., 2021; Fujiki et al., 2008; Griffiths et al., 2020; Löytömäki et al., 2020; Merkenschlager et al., 2012; Spackman et al., 2005; Rieffe & Wiefferink, 2017), emotional functioning (e.g., Conti‐Ramsden et al., 2013; Smit et al., 2019; van den Bedem et al., 2018) and social cognition and responsiveness (e.g., Fujiki et al., 1996; Marton et al., 2005; St Clair et al., 2011). These abilities are vital for a lifetime of healthy interactions with other children and adolescents and are essential for full participation in our society (e.g., Gough Kenyon et al., 2022).

### The implicit learning deficit hypothesis

Given that social‐communicative abilities are to a large extent attained automatically or implicitly, without an explicit intention to learn, the Procedural Deficit Hypothesis (PDH; Ullman & Pierpont, 2005) states that the social‐communicative problems in children with DLD are largely based on a reduced implicit learning ability. Implicit learning refers to the learning of information in an unintentional or incidental way, without awareness of what has been learned (e.g., Cleeremans et al., 1998). However, previous research does not unequivocally confirm worse performance of individuals with DLD on tasks assumed to measure implicit learning (e.g., see Lammertink et al., 2017 and Obeid et al., 2016, vs. West et al., 2021, for meta‐analytic reviews providing positive and no clear evidence, respectively). Moreover, evidence for the assumption of a significant link between performance on implicit learning tasks on one hand and language impairments in DLD on the other is also inconclusive (West et al., 2021). Finally, to our knowledge, there are no previous studies that provide direct evidence for the assumed association between implicit learning, as assessed with any of the common implicit learning tasks, and emotion recognition and social cognition/responsiveness, in TD and/or DLD populations (but see Lieberman, 2000, for indirect evidence).

The mixed results regarding the link between implicit learning and language abilities may at least be partly due to impreciseness in the definition of concepts, and the multitude of tasks that are used to cover them (e.g., Bogaerts et al., 2021). Specifically, the terms procedural learning, statistical learning, sequence learning and implicit learning are often treated as interchangeable concepts, although they may mean different things to different authors. For example, the term procedural learning is sometimes reserved to exclusively refer to tasks in which a cerebellar and/or basal‐ganglia‐associated motor system plays a dominant role, like in the commonly used Serial Reaction Time Task (SRTT), whereas it may also be used to more generally refer to the concept of implicit learning. Regarding ‘implicit learning tasks’, next to the SRTT, other (fairly) commonly used tasks are the Artificial Grammar Learning (AGL) task, the Hebb serial order task, probabilistic category tasks (like the weather prediction task) and contextual cuing tasks (see e.g., West et al., 2021, for a brief overview of the content of these tasks). To further complicate issues, each of these tasks may be presented in different modalities and/or use verbal or non‐verbal stimuli. For example, in their meta‐analyses, Lammertink and colleagues found evidence for impaired implicit learning in auditory statistical learning tasks (e.g., AGL tasks) (Lammerink et al., 2017), but not in visual statistical learning tasks (Lammertink et al., 2020b). These findings suggest that the implicit learning deficit is domain‐specific rather than domain‐general. Finally, tasks may differ in the extent of a potential (compensatory) involvement of explicit learning (e.g., Ullman & Pullman, 2015). Even if, in the best case, all these different tasks and task versions tap into a common (implicit) learning process, they also very likely involve at least partly different cognitive operations and underlying brain structures. These different processes may play a more or less important role in the different types and aspects of social‐communicative abilities, thereby causing much variation in the extent to which implicit learning task performance is predictive of indices of social‐communicative abilities.

For these reasons, we used the somewhat more general term implicit learning deficit (ILD) hypothesis rather than PDH. By doing so, we expressed our aim to expand the hypothesis to also include paradigms that are not (solely) based on the learning of patterns of motor responses, as in the SRTT which is the prototypical ‘procedural’ learning task.

### Associative learning

All or most of the ‘regularity extraction’ tasks used to assess implicit learning are tasks involving the incidental learning of some sequential pattern (e.g., Gerken et al., 2021). Importantly, one cognitive process that may underly performance on all these tasks is associative learning (see Yeaton et al., 2023, for a recent example of this notion; also see Boyer et al., 1998). For example, in the SRTT and AGL task, participants can unintentionally learn associations between adjacent and non‐adjacent elements of the sequence, which in turn are responsible for faster responding on trials that correspond to those associations than on those that do not (e.g., Remillard, 2008). Likewise, performance on contextual cuing tasks is governed by an association between the context that is created by a certain array of distractor stimuli and the location of to‐be‐detected target stimuli. After learning, upon presentation of the context cue, attention is automatically directed to the target location, thereby facilitating fast responding in visual search tasks (e.g., Sisk et al., 2019). If associative learning indeed is the critical cognitive process underlying performance on the commonly used implicit learning tasks, an optimal task to assess whether there is a domain‐general implicit learning deficit in individuals with DLD would require a task with two features. First, the task should explicitly tap into the associative learning process. Second, the task should not place a heavy demand on other processes, such as motor, perceptual, social and linguistic processes.

### Present study

Given the foregoing, in the present study, we used a relatively novel incidental contingency learning task that has been shown to be a reliable measure of associative learning ability. Explicit awareness of the contingencies appears not to be a prerequisite for behavioural performance in this task that is indicative of having learned the contingencies. Moreover, unlike common implicit learning tasks, the contingency learning task does not involve the learning of *sequences* of stimuli, but rather the learning of within‐stimulus feature associations. Finally, our task involves the use of stimuli that may be less directly related to the language deficits implied in DLD, such as is the case when using auditorily‐ or visually‐presented (non‐)words or letters. Collectively, the use of this task allowed us to further assess the proposed domain‐general implicit learning deficit in DLD: will individuals with DLD also show a deficit on a non‐sequential implicit learning task with coloured geometrical stimuli?

We tested whether children with and without DLD differ in performance on the contingency learning task. Moreover, we assessed the extent to which performance on this task is predictive of various aspects of social‐communicative abilities, specifically facial emotion recognition ability, language ability (comprehension, production) and social responsiveness. If the ILD hypothesis is valid, children with DLD should display worse learning in the contingency learning task in comparison to TD children. Moreover, task performance should be significantly related with the various social‐communicative skill measures in one or both groups of children. However, if deficits in implicit learning are restricted to sequential learning tasks that strongly involve motor and/or language processes, then the children with DLD should display no impairment in learning the contingencies. Moreover, this could also imply no strong link between performance on the learning task and the various social‐communicative skill measures.

The outcome of this study may have clinical implications for the feasibility of using interventions that are based on incidental or implicit learning. Specifically, if implicit learning is found to be intact, this would encourage the future development of interventions that are not (only) based on explicit instruction and feedback, but on relatively effortless incidental and implicit learning.

## MATERIAL AND METHODS

### Participants

The participants were 52 (19 boys; 33 girls) children without a DLD diagnosis (TD children), with a mean age of 10.7 years (SD = 0.8; range = 9–13), and 60 (37 boys; 23 girls; mean age: 10.8, SD = 0.9; range = 9–13 children with a DLD diagnosis. The children with DLD were pupils from six special primary schools for children with DLD; the TD children were students from five regular primary schools in different regions of the Netherlands. The DLD diagnosis was established by a multidisciplinary team at an audiological centre. Criteria were (1) no significant hearing issues, determined by an audiologist; (2) typical nonverbal intelligence, assessed by a psychologist; (3) absence of neurological problems; and (4) experiencing severe and persistent language difficulties that negatively affect communication effectiveness (SIAC, 2023). The severity of language difficulties was confirmed by scores on diverse language tests, including the Clinical Evaluation of Language Fundamentals (CELF) and Peabody Picture Vocabulary Test (discussed later), that were 1–2 SDs below average. The persistence of the language problems was typically evaluated yearly by a speech therapist. All children participated voluntarily and received a small present after completing the tests. Written informed consent was obtained from both the school directors and one of the children's parents or caregivers (hereafter referred to as parent). The study was approved by the ethics committee of Radboud University (#ECSW‐2021‐155).

### Instruments

*Contingency Learning Task (CLT)*. This task was programmed in PsychoPy (Peirce et al., 2019) and was based on research by Lin and McLeod (2018) and Schmidt and De Houwer (2012). The task has been found to be reliable (McLeod, 2019). The task started with a 30‐trial practice phase. Each trial commenced with a 250 ms white fixation cross, followed by a string of five identically‐coloured asterisks, displayed in the middle of a black screen. The colours used were red, yellow and green. By pressing one of three keyboard keys (J = red, K = yellow and L = green), the participant had to indicate the colour of the asterisks as fast and as accurately as possible. The asterisks were displayed until the participant made a response or until 2000 ms had passed, whichever came first. Feedback was given in case the participant made an incorrect response or was too late in responding. Feedback consisted of three white X's, displayed in the middle of the screen for 1 s, which was immediately followed by the next trial. The next trial was presented immediately after the previous trial in case of a correct response (no feedback). Each colour was randomly presented 10 times.

**FIGURE 1:**
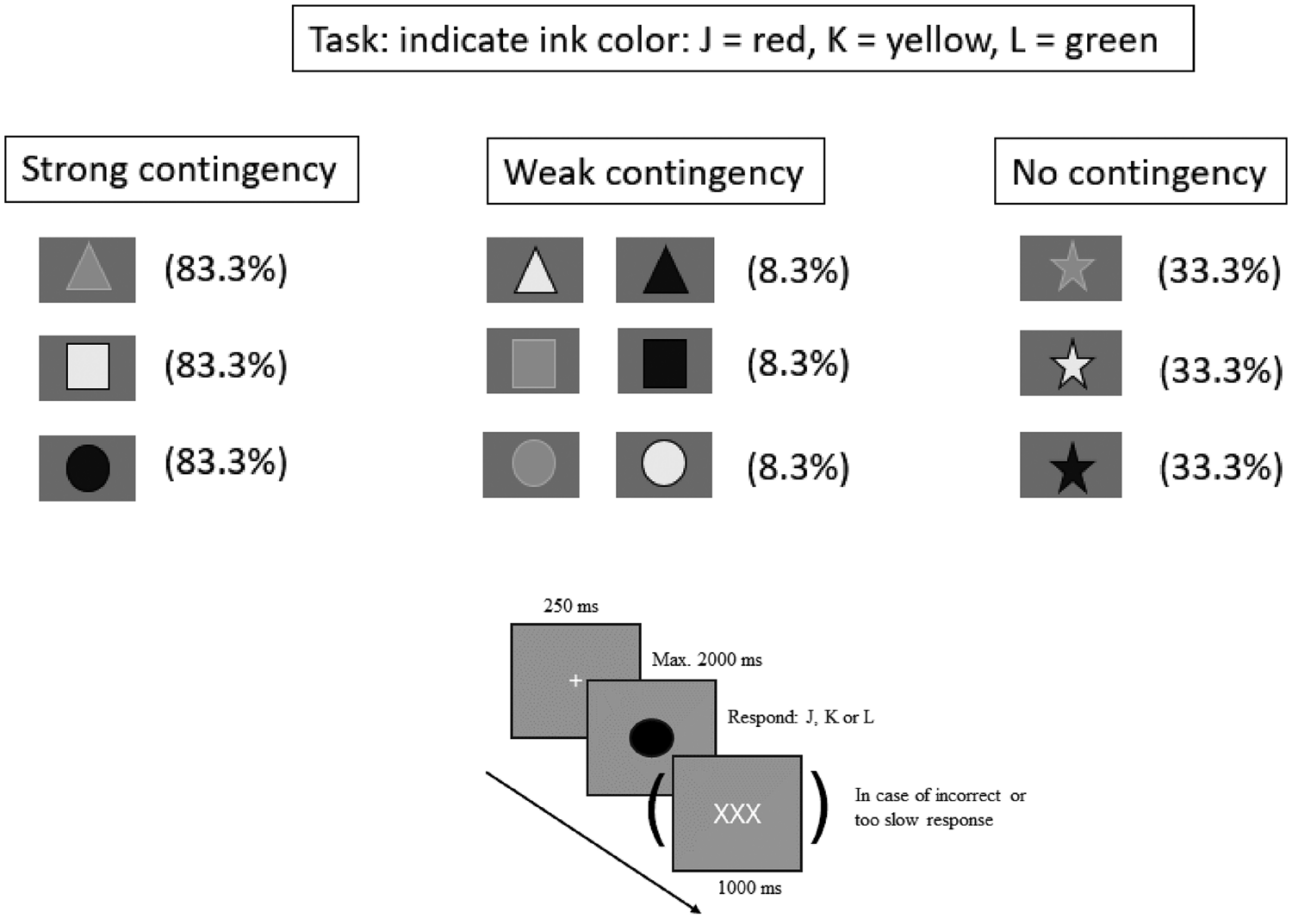
Top: figure‐colour contingencies in one version of the contingency learning task (see text for a detailed task description). Different colours are represented as greyscales: white = yellow; grey = red; black = green. Bottom: timeline of example trial.

The experimental phase was initiated after an instruction screen. During each trial of the main task, the participant was shown a coloured geometric figure. The figures used were a triangle, circle, square and star, which could be red, yellow or green (see Figure 1). As in the practice phase, the participant had to indicate the colour of each figure as quickly as possible by pressing one of three buttons. The children were not informed of the fact that they would see different shapes. Therefore, colour was the relevant dimension whereas shape was irrelevant. However, three of the four figures (maximal duration: 2000 ms) were shown in a certain colour on most (83.3%) of the trials. These were the strong contingency trials. On weak contingency trials (8.3%), each of these figures was presented in one of the other two colours. Finally, on the no contingency trials, the fourth figure was shown in one of the three colours in 33.3% of the cases.

Each participant was assigned to one of four task versions (counterbalanced). Across task versions, each of the four figures functioned as ‘no contingency’ figure and each specific figure occurred in one of the colours an equal number of times. This ensured complete counterbalancing across the task versions of specific figure‐colour combinations and their assignment to the three trial types. The task consisted of four 48‐trial blocks, each consisting of 30 strong contingency trials, 6 weak contingency trials and 12 no contingency trials (see Supplementary Material S1, for a concrete list of stimuli and corresponding trial type used during each trial block, for one version of the CLT). All further details were as described for the practice phase. After the actual experimental trials, the participant received a question asking whether he/she had noticed that some of the figures were shown in a particular colour most of the time (answer options: yes/no). Thereafter, the participant was shown each of the geometric figures, which was accompanied by the question: ‘Guess in which colour this figure was shown most of the time; press “J” for red, “K” for yellow and “L” for green’. After each choice, the participant was also asked to indicate how certain he/she was of the answer just given, using a scale from 1 = very unsure to 4 = very sure. Task completion took about 10 min. The primary dependent measure was the response time (RT) of pressing the correct key. Based on the answer to the questions asked after the experimental trials, an ‘awareness score’ was computed for each participant (hereafter referred to as CLT‐Awareness score). This score was computed by adding, for each correctly identified colour of the three figures that were used as strong and weak contingency trials, the indicated level of certainty about the given answer (min‐max score: 0–12, with a high score indicating strong awareness).

Previous studies with students showed that soon in the course of the task, the strong contingency trials yield faster RTs compared to the weak and no contingency trials, and the weak contingency trials yield larger RTs than the no contingency trials. Putatively, the participant largely unconsciously and automatically learns the association between a geometric shape and the corresponding contingent colour. Hence, according to the example in Figure 1, a triangle will facilitate or ‘prime’ pressing the ‘red’ button. In contrast, this figure will slow down the retrieval of the low contingency colours yellow and green, that is, prompt a longer RT relative to the RT belonging to the no contingency trials. It is assumed that the influence of these associations is largely unconscious due to the lack of time to consciously and intentionally use the knowledge about the contingencies (e.g., that a triangle is usually associated with the colour red): the colour is identified and used to select the response button faster than that the shape of the figure is consciously identified and/or labelled and used for this purpose.

*Emotion Recognition Task (ERT)*. Briefly, this normed and validated task (Kessels et al., 2014) entails the presentation of short video fragments. Each fragment starts with a neutral facial expression, which gradually changes to expressing one of the six basic emotions (anger, sadness, disgust, surprise, joy or fear) to a certain intensity. The different intensities of the morphed facial expressions were 40%, 60%, 80% and 100%. On each trial, the participant was asked to indicate which emotion is displayed, by choosing one of six response alternatives shown to the left of the face. There were four trials for each intensity and emotion, for a total of 96 trials. The participants needed about 10 min to complete this task. The dependent measure was the number of trials with a correct response (min‐max score: 0–96), with a higher score representing a better facial emotion recognition ability.

*Social Responsiveness Scale‐2 (SRS‐2)*. The Dutch translation of the SRS for children (Constantino & Gruber, 2015) was used as a measure of different aspects of social‐communicative abilities. This questionnaire was originally designed to screen for social‐communicative limitations in 4‐ to 18‐year‐old children with autism spectrum disorder. The questionnaire consists of 65 items, which are completed by a parent of the child. The parent must indicate, using a 4‐point answer format (1 = not true; 2 = sometimes true; 3 = often true; 4 = almost always true) the extent to which the information conveyed in each statement is true when considering the child's behaviour over the last 6 months. The instrument consists of five subscales: social awareness (e.g., ‘Expressions on his or her face don't match what he or she is saying’), processing of social information (social cognition; e.g., ‘Recognizes when something is unfair’), competence in mutual social communication (social communication; e.g., ‘Avoids eye contact or has unusual eye contact’), social fear/avoidance (social motivation; e.g., ‘Seems self‐confident when interacting with others’), and restricted interests and repetitive behaviour (e.g., ‘Has repetitive, odd behaviours such as hand flapping or rocking’). Completion of this scale takes about 15–20 min. The total raw score (17 items are reverse scored) was used as an outcome measure (min‐max score: 65–260), with a higher score indicating worse social‐communicative abilities.

*Clinical Evaluation of Language Fundamentals* *(CELF)*. The Dutch version of the CELF (versions 4 and 5; Wiig et al., 2013) was used to assess basic language and communication abilities. The CELF is a test battery for 5‐ to 18‐year‐old children and includes tests measuring language comprehension, production, content and form. The battery is administered by a trained professional (mostly speech‐language therapist). Via the special education schools, we were able to collect data of a reasonable number of children with DLD on eight subscales: (1) recalling sentences, (2) formulating sentences, (3) following directions, (4) understanding spoken paragraphs, (5) word categories, (6) composing sentences, (7) semantic relationships and (8) word definitions. Each subscale contains 20–41 items, which are scored on a 0–3 or 4‐point scale, also depending on whether the fourth or fifth version of the test is used. Subscales 3, 5 and 7 assess receptive language abilities; subscales 1, 2, 5 and 8 cover expressive language. Language content is measured with subscales 3, 4, 5 and 8, whereas language form is assessed with subscales 1, 2 and 6. Administration of all tests takes about 60–90 min. We used the raw score on each subscale as the outcome measure, with a higher score representing a better skill. Note that due to the slight differences between test versions, and the fact that children differed in whether they received version 4 or 5 (for one child, even some subscales were from version 4, whereas the others were from version 5), the total scores are not directly comparable, implying that the score on this measure could only be used as a rough estimate of the child's language abilities.

*Peabody Picture Vocabulary Test (PPVT‐III)*. A Dutch version of the original test (Dunn & Dunn, 1997) was used as (additional) measure of receptive language abilities, specifically vocabulary. On each trial, the participant is asked to match, by pointing, a spoken expression (e.g., ‘Which picture shows laughing?’) to one of four pictures (the distractor pictures display persons involved in other activities). There are 204 items divided into 17 sets of 12 items each. The complexity of the task increases across sets and trials. Based on the age and/or number of errors within a set, a ‘start’ and ‘final’ set of items is determined for each individual participant. Specifically, the starting set is the most complex set for which the participant does not make more than four errors; the final set is the first set in which the participant makes nine or more errors. The raw score is computed by subtracting the total number of errors from the item at which the test is terminated. The test takes 10–15 min to complete.

### Procedure

After obtaining written consent for participation in the research by the school directors, the directors were asked to distribute an information leaflet to the children, who in turn were asked to give this to their parent(s). In case the parent was interested, he or she received more detailed information about the research, including a consent form, via email. After receiving the signed consent form from the parent, further details were provided about the data collection at the school. The parent was asked to fill in the SRS‐2 via Qualtrics software (Qualtrics, 2019); the CELF and PPVT data were collected via the special education schools. The children were individually tested in a separate, quiet school room using a Dell laptop with a 14‐inch screen. The CLT was presented first, followed by the ERT. The SRS‐2 and behavioural test data were collected on about the same date. The CELF data were collected between 134 weeks before and 11 weeks after the children completed the CLT and ERT.

### Data‐analysis

All analyses were performed using SPSS‐29 (IBM, 2023) and JASP (0.18.3; JASP Team, 2024) software. Regarding the RT analysis of the CLT, all trials with an incorrect response (3.89% of the trials) and/or an RT < 200 ms (0.01%) were removed. Based on the remaining trials and a criterion of >3 times the interquartile range, outlying data for each participant (in total 1.38% of the data) were removed. A repeated measures analysis of variance (rm‐ANOVA) was performed with group (DLD vs. TD) as between‐subject factor, trial type (strong, no, vs. weak contingency) and trial block (1–4) as within‐subject factors, and RT as dependent measure. A Greenhouse‐Geisser correction was used in case of violation of sphericity. The overall mean proportion of trials with a correct response was high (0.958), and a Group × Trial Type rm‐ANOVA on the proportion of correct trials only revealed a significant main trial type effect, *p* = 0.02. This reflected a higher accuracy for the strong contingency trials (0.965) than for the no contingency trials (0.956), which yielded a similar proportion of correct responses as did the weak contingency trials (0.953; repeated contrast analysis). All further analyses were based on the RT data. An ANOVA with group as a single factor was performed on the CLT awareness, ERT and SRS‐2 total scores.

Subsequently, we performed correlation analyses, computing the Pearson correlation coefficient between the CLT outcome measures on one hand, and the outcome scores of the ERT, SRS‐2 and the CELF on the other. For the CLT, we computed the difference in RT between weak and strong contingency trials as an outcome measure (hereafter referred to as CLT‐Diff score). A high score represented strong incidental learning. For the ERT and SRS‐2 scores, the correlations were computed for the combined and separate DLD and TD groups. We also computed correlations with each of the five SRS‐2 subscale scores, but the (null) results found when only using the total score did not change. We only reported the correlations with the SRS‐2 total score in the Results section. In both the ANOVAs and the correlation analyses, we used *α* = 0.05 as a criterion for statistical significance.

Concerning the CLT RT analysis, a prior power analysis using GPower (Faul et al., 2007) revealed a group size of 28 to detect the critical group × trial type interaction in a rm‐ANOVA (pooling across the trial block factor) with two groups, three repeated measures, an intermediate effect size of *f* = 0.25, *α* = 0.5, a power of 1‐*β* = 0.80 and an assumed correlation of *r* = 0.5 among the repeated measures. Power analysis for the ERT and SRS‐2 data revealed a sample size of 128 to detect a difference between the two groups, with *f* = 0.25, *α* = 0.5 and 1‐*β* = 0.80. Finally, regarding the correlation analyses, power analysis revealed a sample size of 67 for detecting a correlation of *ρ* = 0.3, again with *α* = 0.5 and 1‐*β* = 0.80. These results imply a sufficiently powered study for the ANOVA on the CLT and ERT data. However, the response rate concerning the SRS‐2 and the CELF was relatively low (see Results section). This implies that the study was somewhat underpowered with respect to the correlation analyses involving these measures. Therefore, we also performed Bayesian correlation analyses, in addition to the hypothesis testing Pearson correlation analyses, to quantify the evidence for either the null or the alternative hypothesis. Here, we used the commonly used classification of Kass and Raftery (1995), with a Bayes factor (BF; BF_01_ = evidence for the null [H_0_] hypothesis; BF_10_ = evidence for the alternative [H_1_] hypothesis) of 1–3.2 representing evidence that is not worth mentioning, and a BF between 3.2–10, 10–100 and >100 reflecting substantial, strong and decisive evidence, respectively.

## RESULTS

### ANOVAs

**FIGURE 2:**
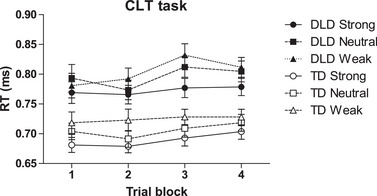
Mean RT (±SEM) of the DLD and TD groups for the different trial types across the four trial blocks of the CLT. S, N and W represent strong, neutral and weak contingency trials, respectively. Abbreviations: CLT, contingency learning task; DLD, developmental language disorder; RT, response time; TD, typically developing.

Figure 2 shows, for each trial block, the mean RT for the different trial types of the CLT, separately for the DLD and TD groups. The data suggest overall slower responding in the DLD compared to the TD group. However, the two groups displayed the same pattern of responding in this task across trial blocks as a function of the three trial types. Specifically, on most trial blocks, the strong contingency trials were associated with the shortest RTs, followed by the neutral trials, and the weak contingency trials, which elicited the longest RTs.

Table 1 presents the statistical details of the outcome measures in the social‐communicative and linguistic domains. The response rate for the SRS‐2 was 35.0% and 59.6% for the parents of the children with DLD and the TD children, respectively. CELF data could be collected for 45.0%–68.3% of the children with DLD, depending on the specific subscale.

**TABLE 1:** Descriptive groups’ details of the social‐communicative and linguistic variables.

		DLD	TD
Instrument	Outcome measure	Mean (SD); min‐max	Mean (SD); min‐max
ERT	#correct	49.22 (9.29); 23–70 (*n* = 60)	53.35 (7.16); 32–71(*n* = 52)
SRS‐2	Total score	140.10 (22.70); 95–172 (*n* = 21)	107.16 (30.13); 71–201 (*n* = 31)
CELF			
‐ RS	Raw score	33.28 (9.32); 16–59 (*n* = 40)	
‐ FS	Raw score	20.64 (8.56); 1–38 (*n* = 39)	
‐ FD	Raw score	21.21 (11.08); 0–47 (*n* = 41)	
‐ US	Raw score	12.79 (5.73); 0–28 (*n* = 39)	
‐ WC	Raw score	19.38 (5.14); 5–29 (*n* = 37)	
‐ CS	Raw score	7.49 (5.15); 0–18 (*n* = 37)	
‐ SR	Raw score	7.15 (4.66); 0–17 (*n* = 27)	
‐ WD	Raw score	5.21 (4.67); 0–21 (*n* = 38)	
PPVT	Raw score	103.05 (12.67); 74–136 (*n* = 40)	

Abbreviations: CELF, Clinical Evaluation of Language Fundamentals; CS, composing sentences; DLD, developmental language disorder; ERT, emotion recognition task; FD, following directions; FS, formulating sentences; PPVT, Peabody Picture Vocabulary Test; RS, recalling sentences; SR, semantic relations; SRS‐2, Social Responsiveness Scale 2; TD, typically developing; US, understanding spoken paragraphs; WC, word categories; WD, word definitions.

The data suggest weaker emotion recognition and general social‐communicative abilities for the DLD than the TD group.

These impressions were confirmed by statistical analyses. Analysis of the RTs of the CLT revealed a significant main effect of group, *F*(1, 110) = 18.99, *p* < 0.001, *η_p_* ^2^ = 0.15, reflecting slower responding in the DLD group (*M* = 0.79; SD = 0.12) than in the TD group (*M* = 0.71; SD = 0.08). The main effect of trial type was also significant, *F*(2, 220) = 30.75, *p* < 0.001, *η_p_* ^2^ = 0.22. Repeated contrast analysis revealed that the children responded faster on the strong contingency trials (*M* = 0.73, SD = 0.11) than on the no contingency trials (*M* = 0.75, SD = 0.12), *t*(110) = −5.32, *p* < 0.001, which in turn elicited faster responding than the weak contingency trials (*M* = 0.77, SD = 0.11), *t*(110) = −2.80, *p* = 0.006. The main trial block effect was also significant, *F*(2.23, 244.8) = 4.80, *p* = 0.007, *η_p_* ^2^ = 0.04. However, subsequent contrast analysis revealed a significantly slower response only during trial block 3 (*M* = 0.76, SD = 0.12) than during block 2 (*M* = 0.74, SD = 0.12), *t*(110) = −3.71, *p* < 0.001. None of the interaction effects were significant, *F*s < 0.14, *p*s > 0.33.

ANOVA on the ERT data revealed significantly fewer correctly recognised facial expressions in the DLD than the TD group, *F*(1, 110) = 6.78, *p* = 0.01, *η_p_* ^2^ = 0.06. Analysis of the SRS‐2 data revealed a significantly higher score, reflecting more social‐communicative problems, for the children with DLD compared to the TD children, *F*(1, 50) = 18.08, *p* < 0.001, *η_p_* ^2^ = 0.27, BF_10_ > 100.

### Correlation analyses

The correlations between the CLT‐Diff and CLT‐Awareness scores on one hand and the ERT, SRS‐2, CELF and PPVT scores on the other, were all not significant. For all correlations, there was either substantial evidence for the null, or evidence for neither the H_0_ nor the H_1_ (see Supplementary Material S2, for further details).

## DISCUSSION

The present study compared the performance of children with and without DLD on a relatively new incidental associative learning task, the CLT. Moreover, we assessed the association between performance on this task and diverse measures of social‐communicative abilities. We found that, in the learning task, both groups of children displayed a pattern of responding that was indicative of incidental learning of the stimulus‐stimulus contingencies implied in the task. However, the correlation analyses largely revealed insignificant correlations.

### Implicit learning in DLD versus TD participants

The present study is the first to use the CLT in children, and the results are similar to those obtained in studies examining adult participants (MacLeod, 2019), including the finding that learning proceeded rather quickly (e.g., Lin & MacLeod, 2018). Moreover, we found evidence that learning in this task was largely implicit. Specifically, the level of awareness of the colour‐figure contingencies was not related to the level of learning those contingencies. If anything, in the DLD group, lower levels of awareness tended to be associated with a larger difference in RT between strong and weak contingency trials (indicative of strong contingency learning; correlation between CLT‐Awareness and CLT‐Diff score: *r* = −0.22; data not shown).

Of primary importance is the finding that, despite their overall slower response in the CLT, the children with DLD showed an equal level and speed of implicit learning as did the TD children. This finding adds to previous results suggesting that DLD is not associated with a *general* implicit learning deficit. Specifically, as also indicated in the introduction, the results of the meta‐analysis by Lammertink et al. (2017), reporting evidence of impaired performance on implicit learning tasks in DLD, were based on tasks exclusively using auditory verbal stimuli. West et al. (2021) found a reliable difference between DLD and TD participants only for studies adopting AGL or weather prediction tasks. Presumably, worse performance on the (auditory) AGL tasks could be explained by the fact that these tasks mainly rely on language abilities and less on implicit learning abilities. Also, the weather prediction task has been criticized for tapping into explicit, declarative memory as well as verbal labelling abilities, rather than into implicit learning (Fotiatis & Protopapas, 2014).

In their meta‐analysis, Obeid et al. (2016) reported that participants with DLD display deficits in statistical learning relative to age‐matched controls, with the majority of the included studies using the SRTT with digits or pictures. These tasks may be argued to not share features with language abilities as much as is the case for the tasks using auditory stimuli and AGL tasks mentioned in the previous paragraph. However, as noted in the introduction, the SRTT involves a sequential stimulus presentation. The results reported by Gerken et al. (2021; see also Hsu & Bishop, 2014) suggest that impaired performance in individuals with DLD is limited to sequential learning tasks, implying a sequential learning pattern deficit rather than a general procedural learning deficit. The results of the present study examining within‐stimulus associations support this suggestion.

However, one exception to this is a very recent study (published before the design and data collection of the present study). Specifically, Derawi et al. (2024) used nonspeech sounds in categorisation tasks. They found that children with DLD, compared to TD children, performed worse in a version of this non‐sequential and non‐linguistic (albeit auditory) task that has been shown previously to depend on implicit learning. Possibly, impaired implicit learning is largely restricted to tasks using auditory stimuli and/or to more complex tasks than simple associative learning tasks.

### Association between implicit learning ability and language abilities

For both children with and without DLD, we found no evidence for an association between performance on the implicit learning task and language abilities. In fact, Bayesian analyses revealed evidence for the null for the majority of the correlations. Inconclusive evidence has also been found in previous studies for the association between performance on: (1) a visual statistical learning task using cartoon drawings and meaningless shapes and tests of written language abilities (Lammertink et al., 2020a), (2) a more common visual SRTT and tests of expressive grammatical proficiency (Lammertink et al., 2020b) and (3) an auditory verbal statistical learning task and tests of expressive knowledge of grammatical rules (measured by the CELF subscales sentence recall and word structure; Lammertink et al., 2020c). In their meta‐analysis, West et al. (2021) found a negligible and nonsignificant pooled correlation between SRTT performance and language abilities, including knowledge of syntactic structure and morphology, receptive grammar, vocabulary, picture‐word matching, spelling and reading ability. However, they did find a modest but significant overall correlation between performance on AGL(‐like) tasks and language abilities, with the majority of studies assessing reading ability.

All in all, the present inconclusive or null results regarding CLT performance and language abilities largely match those obtained in previous studies using more traditional implicit learning tests. The collective results strongly suggest no general implicit learning involvement in learning language‐related abilities in children with and without DLD.

### Association between implicit learning and facial emotion recognition ability

Presumably, learning to recognize the meaning of (more‐or‐less subtle) facial emotional expressions is largely based on implicit learning. Previous studies indeed found evidence for implicit processes involved in learning about faces. For example, Blessing et al. (2010) found that severely amnestic individuals (patients with dementia) were still able to implicitly learn to associate written emotional content to faces. However, we are not aware of any studies directly examining whether or not individual differences in performance on implicit learning tasks are associated with differences in facial emotion recognition ability. Our results suggest a negative answer: despite a significant difference in emotion recognition ability between children with versus without DLD, there was no group difference in CLT performance. Moreover, our correlational analysis also revealed null results. Arguably, this ability reflects an innate capacity, is largely learned in an explicit way (see e.g., Fernández‐Dols & Crivelli, 2015, for discussions about the ‘innate vs. learned’ issue), and/or is largely based on implicit learning processes that are not captured by the CLT (see also subsequent discussion).

### Association between implicit learning and social responsiveness

To the best of our knowledge, only one study explicitly examined the association between implicit learning task performance and social competencies as measured with a social responsiveness scale. Specifically, Zwart et al. (2018) found that performance on a probabilistic SRTT was not associated with the SRS (adult version) total score, in either adults with or without an autism spectrum disorder. The present results add to these null findings by showing that implicit learning, as measured with the CLT, is not associated with the total SRS score in children with and without DLD.

Possibly, social‐communicative abilities are based on implicit learning associations that are much more complex and even more ‘hidden’ than is the case in tasks like the SRTT and the CLT (a suggestion also put forward by Zwart et al., 2018). For example, the interpretation of emotional facial expressions and the understanding of complex social rules or norms may require the learning of conditional relations (e.g., see Battu, 2023). In the framework of emotional face recognition, the meaning of specific cues (e.g., narrowed eyes) may depend on (be conditional on) the presence or absence of other cues (e.g., whether or not the nose is raised). Likewise, in learning social norms, the decision to follow a specific rule may depend on the presence or absence of other individuals following that rule (e.g., Battu, 2023). It remains to be examined in future research whether or not the performance on more complex implicit associative learning tasks, implying higher‐order conditional rules, is significantly related to social‐communicative abilities.

### Strengths and limitations

The strengths of the current study are the use of a task that is held to explicitly and reliably tap into the most critical element shared by all implicit learning tasks, namely associative learning. Moreover, the task enables a continuous, real‐time assessment of learning. This characteristic is not shared by implicit learning tasks like the AGL, in which assessment of learning often takes place after exposure to the sequence of stimuli. Typically, the participant is asked to indicate whether new sequences of stimuli follow the grammatical rules or not. This implies that, unlike in the CLT, responding in the ALG may be determined by processes like familiarity, thereby further limiting the process‐pureness of the task. Furthermore, the learning of colour‐shape associations is assumed to be less dependent on linguistic processes than is the case for tasks employing (non‐)words or letters. Putatively, identifying and/or (internal) labelling (if consciously occurring at all) of the stimulus's colour is an easy task, and responding to the colour proceeds faster than verbally labelling (if occurring at all) its shape. The use of these stimuli may have enhanced the purity of the CLT as a general measure of implicit learning ability. Finally, we were able to include a relatively large number of participants in each group, at least with respect to the analyses of the CLT and ERT data.

However, there were also a number of study limitations. The relatively low response rate regarding the SRS‐2 (especially for the children with DLD) and CELF implied a somewhat underpowered study with respect to the analyses involving the corresponding scores. Concerning the SRS‐2, there may be at least two reasons for the lower response rate for the parents of the children with DLD (35.0%) relative to the parents of the TD children (59.6%). First, this group consisted of a relatively large number of parents for whom Dutch was not their native language. Second, this group is relatively frequently asked to participate in research and may feel overloaded. These factors may cause (additional) hurdles to fill in the questionnaire. In addition, the temporal distance between the CLT and CELF scores varied greatly, and the CELF scores were derived from two different test versions. These features negatively impact the validity of the results of the corresponding analyses. The inclusion of Bayesian statistics, next to the regular hypothesis testing statistics, may have partly addressed the power concern, increasing our confidence in the validity of the reported (null) results. However, further research is necessary to derive more decisive conclusions regarding the association between associative learning ability and language abilities.

### Possible implications

Given the intact implicit learning ability of children with DLD, suggested by the past and current results, one may speculate that it is meaningful to consider intervention programs that are based on implicit learning, to enhance their compromised social‐communicative abilities. In general, such programs have the benefit of being less effortful compared to more traditional explicit, trial‐and‐error‐based teaching strategies using explicit instructions and feedback. Implicit, effortless techniques can avert frustration and the learning of incorrect information (e.g., Roberts et al., 2018). Adopting implicit training techniques might be beneficial despite our failure to find reliable associations between implicit learning ability and social‐communicative abilities. As noted previously, social‐communicative abilities might be based on complex, higher‐order, conditional associations that are insufficiently tapped by tasks like the SRTT and the CLT. Importantly, the assumption made here is that also children with DLD are, in principle, able to acquire such associations (given their intact general implicit learning capacity) but that they need more targeted interventions that are preferably based on implicit learning. Two pieces of preliminary evidence speaking to the issue of the viability of implicitly training social‐communicative abilities are provided by Costea et al. (2023), who showed that participants can implicitly learn to regulate the emotional expression of avatars, and Broedelet et al. (2023), who found that children with DLD can implicitly learn to associate novel objects with spoken novel words.

## CONCLUSIONS

The present study revealed no general implicit learning deficit in children with DLD, as evidenced by their performance on an incidental contingency learning task. These children displayed deficits in social‐communicative abilities, specifically language abilities, facial emotion recognition ability and social responsiveness. However, in neither children with DLD nor in TD children was performance on the implicit learning task significantly related to any of the mentioned social‐communicative abilities. These results are not in accordance with the hypothesis that implicit learning deficits underly social‐communicative impairments. However, theoretical considerations and preliminary evidence suggest that interventions directed at improving social‐communicative abilities might profit from incorporating implicit learning strategies.

## CONFLICT OF INTEREST STATEMENT

The authors declare that they have no conflict of interest.

## Supporting information



Supporting Information

## Data Availability

The pre‐processed data are openly available via the Open Science Framework (OSF) under https://doi.org/10.17605/OSF.IO/M35NZ
