# Supplementary material for: Incidental learning and social‐communicative abilities in children with developmental language disorder: Further evaluating the implicit learning deficit hypothesis
Source: Int J Lang Commun Disord. 2025 Feb 20;60(2):e70017. doi: 10.1111/1460-6984.70017 (PMC11842015; doi:10.1111/1460-6984.70017)
Supplement: Supplementary file 1 — Supporting Information [file JLCD-60-0-s001.docx]

**Supplementary Material**

**S1**. Trial types for one 48-trial block of one of the CLT versions (input file used in corresponding PsychoPy program), S, N, W = strong, neutral, or weak contingency, respectively. On each run, the trials were randomly chosen from the list.

| figure | correctAnswer | contingency | |
| --- | --- | --- | --- |
| GreenCircle.bmp | l | S |  |
| GreenCircle.bmp | l | S |  |
| GreenCircle.bmp | l | S |  |
| GreenCircle.bmp | l | S |  |
| GreenCircle.bmp | l | S |  |
| GreenCircle.bmp | l | S |  |
| GreenCircle.bmp | l | S |  |
| GreenCircle.bmp | l | S |  |
| GreenCircle.bmp | l | S |  |
| GreenCircle.bmp | l | S |  |
| GreenSquare.bmp | l | W |  |
| GreenTriangle.bmp | l | W |  |
| GreenStar.bmp | l | N |  |
| GreenStar.bmp | l | N |  |
| GreenStar.bmp | l | N |  |
| GreenStar.bmp | l | N |  |
| RedCircle.bmp | j | W |  |
| RedSquare.bmp | j | W |  |
| RedTriangle.bmp | j | S |  |
| RedTriangle.bmp | j | S |  |
| RedTriangle.bmp | j | S |  |
| RedTriangle.bmp | j | S |  |
| RedTriangle.bmp | j | S |  |
| RedTriangle.bmp | j | S |  |
| RedTriangle.bmp | j | S |  |
| RedTriangle.bmp | j | S |  |
| RedTriangle.bmp | j | S |  |
| RedTriangle.bmp | j | S |  |
| RedStar.bmp | j | N |  |
| RedStar.bmp | j | N |  |
| RedStar.bmp | j | N |  |
| RedStar.bmp | j | N |  |
| YellowCircle.bmp | k | W |  |
| YellowSquare.bmp | k | S |  |
| YellowSquare.bmp | k | S |  |
| YellowSquare.bmp | k | S |  |
| YellowSquare.bmp | k | S |  |
| YellowSquare.bmp | k | S |  |
| YellowSquare.bmp | k | S |  |
| YellowSquare.bmp | k | S |  |
| YellowSquare.bmp | k | S |  |
| YellowSquare.bmp | k | S |  |
| YellowSquare.bmp | k | S |  |
| YellowTriangle.bmp | k | W |  |
| YellowStar.bmp | k | N |  |
| YellowStar.bmp | k | N |  |
| YellowStar.bmp | k | N |  |
| YellowStar.bmp | k | N |  |

**S2.** Correlations between CLT outcome measures and the ERT, SRS-2, CELF, and PPVT (subscale) scores for the combined and separate DLD and TD groups.

|  | **Total sample** | | **DLD group** | | **TD group** | |
| --- | --- | --- | --- | --- | --- | --- |
| **Measure** | *CLT-Diff* | *CLT-Awareness* | *CLT-Diff* | *CLT-Awareness* | *CLT-Diff* | *CLT-Awareness* |
| ERT | -.04 (*n*=112) | .07 (*n*=112) | -.07 (*n*=60) | .01 (*n*=60) | -.00 (*n*=52) | .12 (*n*=52) |
| SRS-2 | -.03 (*n*=52) | -.10 (*n*=52) | -.18* (*n*=21) | .22 (*n*=21) | .08 (*n*=31) | -.11 (*n*=31) |
| CELF |  |  |  |  |  |  |
| - RS |  |  | -.01 (*n*=40) | .23*(*n*=40) |  |  |
| - FS |  |  | .12 (*n*=39) | .08 (*n*=39) |  |  |
| - FD |  |  | -.01 (*n*=41) | -.12 (*n*=41) |  |  |
| - US |  |  | -.16* (*n*=39) | -.03 (*n*=39) |  |  |
| - WC |  |  | .15 (*n*=37) | .22* (*n=*37) |  |  |
| - CS |  |  | -.05 (*n*=37) | -.09 (*n*=37) |  |  |
| - SR |  |  | .08 (*n*=27) | .08 (*n*=27) |  |  |
| - WD |  |  | -.06 (*n*=38) | .16* (*n*=38) |  |  |
| PPVT |  |  | .21* (*n*=40) | .13 (*n*=40) |  |  |

*Note*. CLT = Contingency Learning Task; Diff = RT difference score; Awareness = awareness score; ERT = Emotion Recognition Task; SRS-2 = Social Responsiveness Scale. CELF = Clinical Evaluation of Language Fundamentals; RS = recalling sentences; FS = formulating sentences; FD = following directions; US = understanding spoken paragraphs; WC = word categories; CS = composing sentences; SR = semantic relations; WD = word definitions. PPVT = Peabody Picture Vocabulary Test. *Bayesian evidence for neither H_0_ nor H_1_ (BF_01_ < 3.2); for all other correlations, there was substantial Bayesian evidence for the null hypothesis. *p* > .05 for all correlations.
